# A Mild Method for Surface-Grafting PEG Onto Segmented Poly(Ester-Urethane) Film with High Grafting Density for Biomedical Purpose

**DOI:** 10.3390/polym10101125

**Published:** 2018-10-10

**Authors:** Lulu Liu, Yuanyuan Gao, Juan Zhao, Litong Yuan, Chenglin Li, Zhaojun Liu, Zhaosheng Hou

**Affiliations:** College of Chemistry, Chemical Engineering and Materials Science, Shandong Normal University, Jinan 250014, Shandong, China; Lulu0214@163.com (L.L.); mgaoyuanyuan@163.com (Y.G.); zhaojuan1016@yeah.net (J.Z.); ylt199710@163.com (L.Y.); chenglinli01@163.com (C.L.); zhaojun0403 @126.com (Z.L)

**Keywords:** segmented poly(ester-urethane), poly(ethylene glycol), surface grafting, chemical treatment, high grafting density, hemocompatibility

## Abstract

In the paper, poly(ethylene glycol) (PEG) was grafted on the surface of poly(ester-urethane) (SPEU) film with high grafting density for biomedical purposes. The PEG-surface-grafted SPEU (SPEU-PEG) was prepared by a three-step chemical treatment under mild-reaction conditions. Firstly, the SPEU film surface was treated with 1,6-hexanediisocyanate to introduce -NCO groups on the surface with high density (5.28 × 10^−7^ mol/cm^2^) by allophanate reaction; subsequently, the -NCO groups attached to SPEU surface were coupled with one of -NH_2_ groups of tris(2-aminoethyl)amine via condensation reaction to immobilize -NH_2_ on the surface; finally, PEG with different molecular weight was grafted on the SPEU surface through Michael addition between terminal C = C bond of monoallyloxy PEG and -NH_2_ group on the film surface. The chemical structure and modified surface were characterized by FT-IR, ^1^H NMR, X-ray photoelectron spectroscopy (XPS), and water contact angle. The SPEU-PEGs displaying much lower water contact angles (23.9–21.8°) than SPEU (80.5°) indicated that the hydrophilic PEG chains improved the surface hydrophilicity significantly. The SPEU-PEG films possessed outstanding mechanical properties with strain at break of 866–884% and ultimate stress of 35.5–36.4 MPa, which were slightly lower than those of parent film, verifying that the chemical treatments had minimum deterioration on the mechanical properties of the substrate. The bovine serum albumin adsorption and platelet adhesion tests revealed that SPEU-PEGs had improved resistance to protein adsorption (3.02–2.78 μg/cm^2^) and possessed good resistance to platelet adhesion (781–697 per mm^2^), indicating good surface hemocompatibility. In addition, due to the high grafting density, the molecular weight of surface-grafted PEG had marginal effect on the surface hydrophilicity and hemocompatibility.

## 1. Introduction

Segmented polyurethane (SPU) is widely used in biomedical fields, such as cardiovascular devices, artificial organs, and tissue engineering scaffolds, due to its long-term bio-stability, excellent mechanical properties, and relatively superior biocompatibility [1,2,3,4,5,6]. However, when SPU is used as long-term blood-contacting materials, the surface of SPU films will result in significant adsorption of proteins, and induce platelet adhesion by activating the coagulation pathway, eventually leading to the formation of microscopic thrombi [7,8]. In addition, the biofouling on SPU surfaces can reduce its mechanical properties [9,10]. To further improve their hemocompatibility, much attention has been paid to producing a nonspecific protein repelling surface by surface modification and creating highly effective non-thrombogenic devices. A preferred strategy is to immobilize natural or synthetic materials onto the hydrophobic surfaces that shield the surface, thus introducing a high activation barrier to repel proteins [11,12,13]. Among them, grafting poly(ethylene glycol) (PEG) onto the SPU surface has attracted considerable interest because PEG can effectively prevent protein adsorption and platelet adhesion mostly due to its low interfacial free energy with water, unique solution properties, hydrophilicity, high chain mobility, and steric stabilization effect [14]. Additionally, much theoretical work is generated to explain the early discovery that grafted PEG chains resist protein adsorption to a high degree [15,16].

Many kinds of surface modification approaches, including chemical treatment, strong oxidation, plasma treatment, UV irradiation, and laser treatment have been used to modify the surface of medical SPU [17,18,19,20]. Among the techniques, chemical treatment possesses some advantageous uniqueness, such as has clearer mechanism and predictable products, and the reaction rate can accurately be controlled by adjusting the reaction parameters. Moreover, the chemical treatment methods including click chemistry [21] and NHS-amine reaction [22] are adopted to modify poly(ε-caprolactone) dendrimer and poly(ethylene-co-acrylic acid) films, respectively. The most commonly used surface chemical treatment to modify the SPU film is allophanate reaction, which is to attack on N-H bonds of urethane groups in the backbone by small molecular diisocyanate to immobilize free -NCO groups on the surface, and subsequently PEG (or PEG derivatives) is grafted on the surface using the chemical reaction [13,23,24]. However, in order to obtain high -NCO density on the surface, the allophanate reaction should be carried out at high reaction temperature of 50–70 °C, which inevitably deteriorates the bulk properties of the substrates. On the other hand, even if using the high reaction temperature, the -NCO density on the surface was unsatisfactory due to the low -NH- content in SPU.

In our previous report [25], a new biodegradable segmented poly(ester-urethane) (SPEU) with uniform-size hard segments was prepared using aliphatic diurethane diisocyanate (1,6-hexanediisocyanate-1,4-butanediol-1,6-hexanediisocyanate, HBH) as a chain extender. The SPEU, which exhibited excellent mechanical properties comparable to MDI-based PU, could meet the requirement of long-term implant biomaterial. The PEG was grafted on the surface of SPEU films via aminolysis. However, the grafting density was only 2.74 × 10^−7^ mol/cm^2^, and the results of protein adsorption and platelet adhesion tests showed that the surface hemocompatibility needed to be further improved. In consideration of there being four urethane groups in each hard segment, the SPEU possesses higher −NH- content than MDI-based PU, and thus, the high grafting density on the film surface could be obtained by chemical treatment with diisocyanate.

**Figure 1:**
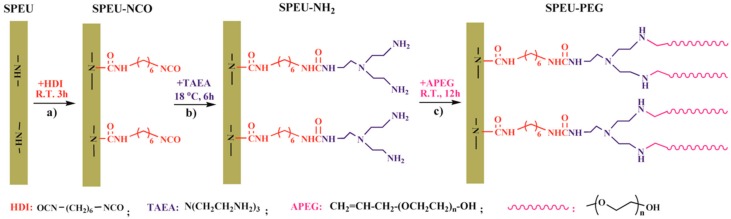
Schematic diagram of grafting PEG onto SPEU surface via (**a**) allophanate reaction; (**b**) condensation reaction; and (**c**) Michael addition reaction.

In this study, we graft PEG on the surface of SPEU film to obtain a high grafting density without deteriorating the intrinsic mechanical properties of the substrates. The surface grafting of PEG on SPEU films was prepared by a three-step chemical treatment (Figure 1), which were all carried out under moderate conditions. The synthetic scheme involved coupling 1,6-hexanediisocyanate (HDI) to the surfaces of SPEU through allophanate reaction (Figure 1a); the -NCO groups attached to SPEU surface (SPEU-NCO) were then coupled -NH_2_ groups of tris(2-aminoethyl)amine (TAEA) via condensation reaction (Figure 1b) to immobilize -NH_2_ on the SPEU surface (SPEU-NH_2_); finally, the PEG was grafted on the SPEU (SPEU-PEG) surface through Michael addition (Figure 1c) between double bond of monoallyloxy poly(ethylene glycol) (APEG) and a primary amino group on the film surface. The chemical structure and modified surface were characterized by Fourier transform infrared spectroscopy (FT-IR), H Nuclear magnetic resonance (^1^H NMR), X-ray photoelectron spectroscopy (XPS), and water contact angle measurements. The influence of chemical treatments on the mechanical properties of the films was researched, and surface hemocompatibility of the PEG-grafted SPEU films was evaluated by protein adsorption and platelet adhesion tests. Furthermore, the effect of molecular weight of grafted PEG on the surface hydrophilicity and hemocompatibility was preliminarily studied.

## 2. Materials and Methods

### 2.1. Materials

Poly(ε-caprolactone) (PCL, *M*_n_ = 2000 g/mol) was supplied by Shenzhen Polymtek Biomaterial Co., Ltd. (Shenzhen, China) and dried for 4 h at 100 °C under vacuum prior to use. HBH was synthesized in our lab according our published paper [4] and the chemical structure was confirmed by ^1^H NMR, ^13^C NMR, and HR-MS. HDI and dibutyltin dilaurate (DBTDL) were purchased from Sigma-Aldrich Chemical Co. (St Louis, MO, USA) and used without further purification. TAEA, (> 97%, Shanghai Macklin Biochemical Co., Ltd. Shanghai, China) was dried over 4-Å molecular sieves and redistilled before use. APEG (*M*_n_ = 1200, 2400, 4000 g/mol) was obtained from Shanghai Aladdin Reagent Co. (Shanghai, China) and was used as received. *N*,*N*-dimethylformamide (DMF, AR grade, Beijing Chemical Reagent Co., Ltd, Beijing, China) was dried with phosphorus pentoxide and distilled under reduced pressure before use. Other reagents were AR grade and purified by standard methods.

### 2.2. Preparation of SPEU and SPEU Films

SPEU was synthesized according to our previous publication [25]. Briefly, the DMF solution of HBH (25 wt %) was added dropwise into the PCL containing DBTDL (0.3 wt % of PCL) with vigorous mechanical stirring under dried nitrogen atmosphere at 80°C. The molar ratio of -NCO/-OH was controlled at 1.02. After that, the reaction mixture was allowed to proceed at the same temperature until the -NCO peak (~2270 cm^−^^1^) in the FT-IR spectrum disappeared (~3.5 h), and subsequently diluted with DMF to approximately 4.5 g/100 mL. The diluted solution was poured into a Teflon mold. The solvent was removed by natural volatilizing at 50 °C for 4 days, and the semitransparent films with 0.20 ± 0.02 mm thickness were subsequently vacuum dried at 40 °C for 1 day to remove the last traces of solvent. GPC (THF): *M*_w_ = 111000, *M*_n_ = 83000, *M*_w_/*M*_n_ = 1.33.

### 2.3. Grafting of PEG on the SPEU Film Surface

Before chemical treatments, the surfaces of the SPEU film discs with ~10 mm diameter were ultrasonically cleaned in 50% ethanol for 30 min and then dried in a vacuum. First, HDI (1.0 g) and DBTDL (0.02 g) were dissolved in anhydrous toluene (10 mL) to get a homogeneous solution, SPEU disc was immersed in the solution and the reaction was allowed to shake for 3 h at room temperature. The film disc was taken out and rinsed with anhydrous toluene to remove unreacted HDI from the surface completely. The SPEU-NCO film achieved was then soaked in 10 mL anhydrous toluene containing 0.13 g TAEA. After gentle shaking at 18 °C for 6 h, the film disc was removed from the solution and flushed with anhydrous toluene to obtain the PEU-NH_2_ film. Finally, the PEU-NH_2_ film was immersed in 10 mL absolute ethanol containing APEG with different molecular weight (0.3 mmol/mL). After gentle shaking for 12 h at room temperature, the films were rinsed with absolute ethanol thoroughly and dried under vacuum at room temperature to constant weight. Three SPEU-PEGs based on APEG with *M*_n_ = 1200, 2400 and 4000 g/mol were named as SPEU-PEG-a, SPEU-PEG-b, and SPEU-PEG-c, respectively.

### 2.4. Instruments and Characterization

FT-IR: FT-IR spectra were recorder on an Alpha infrared spectrometer (Bruker, Germany) equipped with a Bruker platinum ATR accessory in the range of 4000-400 cm^−1^ with the resolution of 4 cm^−1^.

^1^H NMR: NMR spectra were recorded with a 400 MHz Avance II spectrometer (Bruker, BioSpin GmbH, Rheinstetten, Germany) with CDCl_3_ as the solvent.

GPC: The number average molecular weight (*M*_n_) and molecular weight distribution (*M_w_/M*_n_) were measured by gel permeation chromatography (GPC, Waters Alliance GPC 2000). The continuous phase was tetrahydrofuran, and monodisperse polystyrene was used as the calibration standards.

Mechanical properties: Tensile strength properties were determined with a single-column tensile test machine (Model HY939C, Dongguan Hengyu Instruments, Ltd., Dongguan, China) at room temperature. Dumbbell-shaped specimens were punched from the films with a punching die of 12 mm width and 75 mm length, the neck width and length were 4.0 and 30 mm, respectively. The crosshead speed was controlled at 50 mm/min. At least five specimens were measured and the averaged results were reported.

XPS: Chemical composition of the film surface was characterized by XPS (ESCALAB250Xi, Thermo Scientific, Waltham, UK) with a mono-chromated Al-Kα radiation source (energy 1486.68 eV). The base pressure for the measurement was ~3 × 10^−7^ Pa. The binding energies were referenced to the C1s line at 284.8 eV from adventitious carbon. The measurement was carried out at room temperature with a 90° take-off angle of the photoelectron. The atomic concentrations of the elements were determined by their corresponding peak areas.

Water contact angle: The static water-contact angles of blank and modified SPEU films were measured on a drop shape analysis system (CAM 200, KSV Instruments, Helsinki, Finland) at room temperature. Ultrapure water was used as test fluid, and the contact angles were read within 3 s after each drop by using a microscopy. At least six replicate measurements were performed on each specimen and the average was calculated

Protein adsorption: Bradford’s protein determining method was used to assay the albumin adsorbed on the film surface with bovine serum albumin (BSA) as the model protein [26,27]. Before measurement, all the film discs (~10 mm in diameter) were aged in phosphate buffer saline (PBS, pH = 7.4) for one day to remove the physically adsorbed impurities and to achieve complete hydration. The BSA concentration (initial concentration: 1.0 mg/mL) was diluted to 45 μg/mL with PBS. In a typical adsorption experiment, a film disc was immersed in 10 mL of BSA solution at 37°C for 1 h. At the end of the predetermined equilibrium period (1 h), the disc was taken out and rinsed with fresh PBS for several times remove the unbound BSA. The adsorbed protein on the surface was detached in 1 wt % sodium dodecylsulfonate aqueous solution by the stirring method (100 rpm for 1 h). The concentration of the adsorbed BSA was determined with micro-Bradford protein assay method [25,28], and the adsorbed amount was calculated according to the standard curve. The reported values were the average of three independent measurements.

Platelet adhesion: The interaction between the film surface and blood was evaluated by platelet adhesion tests. The platelet-rich plasma (PRP) was prepared from fresh rabbit blood containing sodium citrate as an anticoagulant (Shandong Success Biological Technology Co., Ltd., Jinan, China) according the reported literature [29]. The film discs with ~10 mm diameter were firstly soaked with PBS (pH = 7.4) for 24 h to achieve complete hydrated surface, and then immerged in 1.0 mL PRP and incubated for 60 min at 37 °C. After that, the discs were taken out and rinsed with PBS to remove the non-adherent platelet. Subsequently, the platelets adhering on the surface were fixed with a 2.5% glutaraldehyde for 30 min at 37 °C, and the discs were thoroughly rinsed with PBS and dehydrated by treating with gradual ethanol/water solution from 50% to 100% ethanol (*v*:*v*) with a step of 10% for 30 min in each step. Finally, the platelet-attached surfaces were allowed to dry at room temperature and coated with gold prior to being observed with a Cold Field Emission Scanning Electron Microscope (FE-SEM, Hitachi SU8010, Hitachi, Tokyo, Japan). Different fields were randomly observed. Quantification of adhered platelets was calculated from eight different areas of one specimen using FE-SEM images.

## 3. Results and Discussion

### 3.1. Preparation and Characterization

#### 3.1.1. Activating SPEU Surface with HDI (SPEU-NCO)

**Figure 2:**
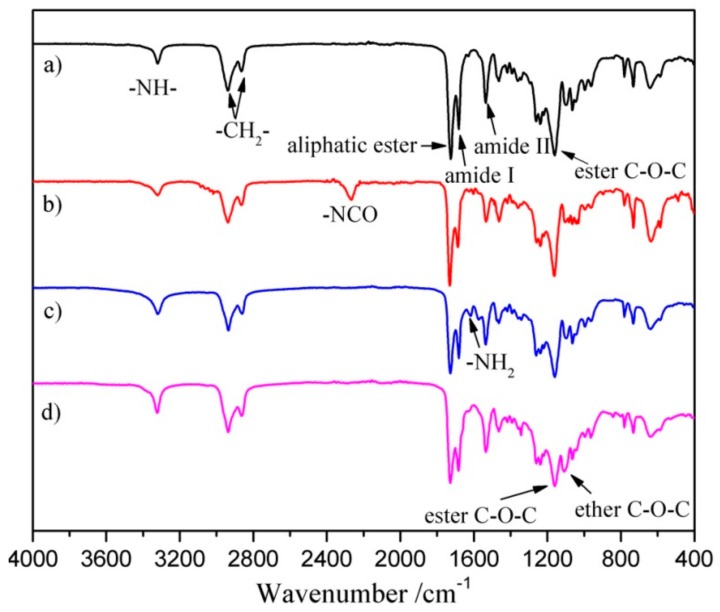
FT-IR spectra of (**a**) SPEU; (**b**) SPEU-NCO; (**c**) SPEU-NH_2_; and (**d**) SPEU-PEG-a films.

The free -NCO groups were grafted onto SPEU film surface by the allophanate reaction (Figure 1a) between -NH- proton of urethane group and -NCO group of HDI in the presence of DBTDL catalyst. The reaction temperature was controlled at room temperature for the purpose of minimum deterioration on the SPEU substrate. The density of free -NCO group on the surface measured by di-n-butylamine back titration method [30] was 5.28 × 10^−7^ mol/cm^2^, which was much higher than that of MDI-based PU (2.5 × 10^−8^ mol/cm^2^, Pellethane^®^, Dow Chemical Co., Midland, MI, USA) in the reported paper in Reference [31]. It was obviously attributed to the high content of urethane (-NH-) groups in SPEU, in which each hard segment contained four urethane groups. The calculated -NH- content in SPEU was 1.67 × 10^−3^ mol/g, while the -NH- content in Pellethane^®^ PU was only 0.9 × 10^−3^ mol/g (the result is based on the same soft segment content in SPEU with Pellethane^®^ PU). After introducing the -NCO groups onto the SPEU surface via allophanate reaction with HDI, a sharp peak at 2270 cm^−1^, the characteristic symmetric stretching vibration peak of -NCO, was observed in the FT-IR spectrum of SPEU-NCO film (Figure 2b), which strongly supported that -NCO groups had been immobilized onto the SPEU film surface.

#### 3.1.2. Introducing -NH_2_ Group onto SPEU-NCO Film (SPEU-NH_2_)

The free -NCO groups on the surface were reacted with -NH_2_ groups of TAEA by condensation reaction (Figure 1b) to introduce -NH_2_ on the film surface. Obviously, one TAEA molecule contains three -NH_2_ groups, two -NH_2_ groups can remain on the surface after one -NH_2_ reacts with the -NCO group, thus producing more -NH_2_ groups on the SPEU surface. However, because of the high reactive activity of -NCO group, the neighboring -NCO groups on surface may react with the -NH_2_ groups in one TAEA molecule, which can reduce the density of -NH_2_ groups on film surface [20]. With the purpose of decrease side reaction, the condensation reaction is allowed at low temperature of 18 °C for 12 h without catalyst. The density of -NH_2_ groups on the film surface, which was determined by an Acid Orange II assay according to reported method [32], was 9.98 × 10^−7^ mol/cm^2^. The result was almost twice as much as that of -NCO groups on the SPEU-NCO film surface, indicating a minimum side reaction. In the FT-IR spectrum of SPEU-NH_2_ (Figure 2c), the significant change is the disappearance of the peak at 2265 cm^−1^, due to -NCO groups completely reacted with -NH_2_ groups of TAEA. Comparing with the FT-IR spectrum of SPEU-NCO (Figure 2b), the absorption intensity of the peak at 3324 cm^−1^, 1678 cm^−1^, and 1529 cm^−1^ increased obviously, which was attributed to the N-H stretching vibration, amide I and amide II of the newly formed ureido. In addition, the characteristic peak at 1611 cm^−1^, a symmetric bending vibration peak of -NH_2_, confirmed the introduction of -NH_2_ on the surface [33].

#### 3.1.3. Grafting of PEG onto SPEU-NH_2_ Film (SPEU-PEG)

**Figure 3:**
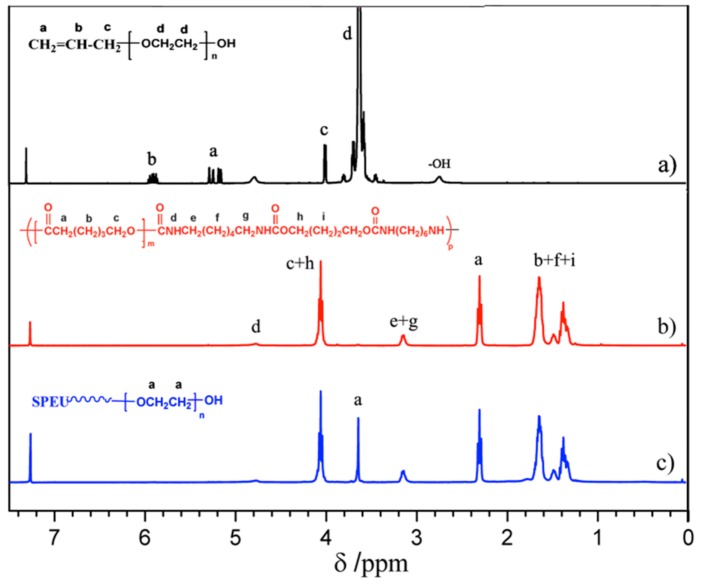
^1^H NMR spectra of (**a**) APEG; (**b**) SPEU; and (**c**) SPEU-PEG-a.

The terminal double bond of APEG reacted with primary amino group on the surface of PEU-NH_2_ film by Michael addition (Figure 1c) to realize grafting PEG onto the PEU film. As we know, Michael addition not only possesses excellent features including mild reaction conditions, high functional group tolerance and high conversions, but also can avoid the use of cytotoxic free-radicals [34]. The SPEU-PEG films were characterized by FT-IR and ^1^H NMR (SPEU-PEGs had the similar spectra), the spectra of SPEU-PEG-a are shown in Figure 2d and Figure 3c, respectively. From the FT-IR spectrum, it could be found that the -NH_2_ absorption peak at 1611 cm^−1^ disappeared thoroughly, and the peak intensity at 3329 cm^−1^ increased slightly which was due to the reaction between -NH_2_ group and APEG. In addition, the characteristic absorption bands at 1166 cm^−1^ and 1095 cm^−1^ belonged to the stretching vibration of ester bonds C-O-C of SPEU and ether bonds C-O-C of grafted PEG. In the ^1^H NMR spectrum, the proton signals of double bond at δ 5.25 and δ 5.92 ppm (Figure 3a) disappeared completely, alongside the proton signals at δ 3.65 ppm belonging to the protons of repeat units of grafted PEG, demonstrating the complete Michael addition. This was another key feature indicating that the PEG was grafted on the film surface.

### 3.2. XPS Analysis

**Figure 4:**
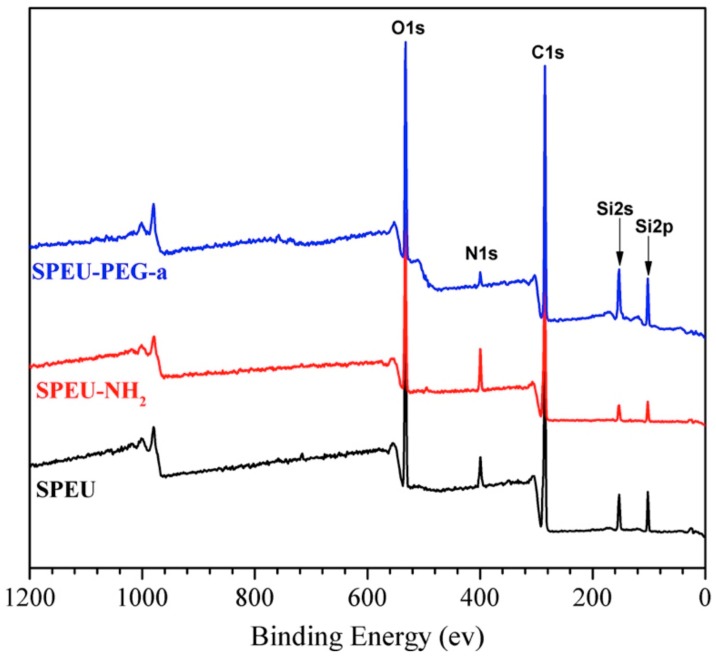
Overview XPS spectra of the SPEU, SPEU-NH_2_, and SPEU-PEG-a films.

The presented XPS investigations were performed in order to extract quantitative results about the surface composition. The overview XPS spectra of the SPEU, SPEU-NH_2_ and SPEU-PEG-a films (SPEU-PEGs had the similar XPS spectra) are illustrated in Figure 4, and the relevant compositions of the surface elements (O1s, C1s and N1s) calculated from XPS results are summarized in Table 1. The blank SPEU film exhibited N1s signal at 398.8 eV with the content of 3.1%, while the N1s signal of SPEU-NH_2_ film increased obviously with the content increasing to 10.3%, which indicated that -NH_2_ groups had been immobilized onto the surface. Compared to SPEU-NH_2_, the nitrogen content of SPEU-PEG decreased significantly, and with the increasing molecular weight of surface-grafted PEG, the nitrogen content decreased gradually. After the PEG is grafted on the surface with high grafting density, the PEG chains can form a thin layer coating on the surface which hinders the inner nitrogen element to be detected, leading to the low surface nitrogen content. The results confirmed that PEG was successfully immobilized onto SPEU surface.

### 3.3. Mechanical Properties

**Figure 5:**
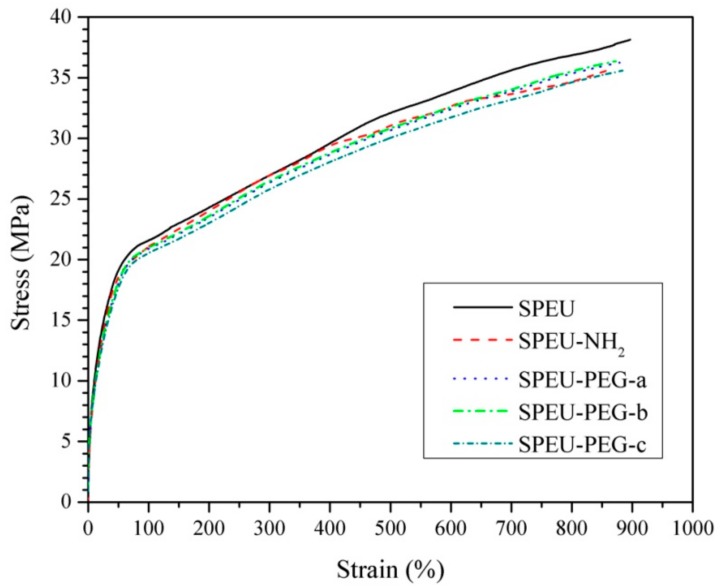
Stress-strain behaviors of blank and modified SPEU films.

Mechanical property was one of the most important properties for long-term implant biomaterials. The stress-strain behaviors of the blank and modified films are displayed in Figure 5 and the corresponding characteristic values derived from these curves are shown in Table 2. Two different regions being visible clearly from the curves manifested that all the films behaved as a soft elastic material, showing a smooth transition in stress-strain behaviors from elastic to plastic deformation regions [35]. The blank SPEU film exhibited outstanding mechanical properties with a strain at break of 896% and an ultimate stress of 38.1 MPa. The excellent mechanical properties should be attributed to the compact physical-linking network structure formed by multiple H-bonds existing among urethane groups and between urethane and ester groups, as per the description in our previous report [36]. The modified films, including SPEU-NH_2_, SPEU-PEG-a, SPEU-PEG-b, and SPEU-PEG-c films displayed analogous stress-strain behaviors with the strain at break of 866–884% and ultimate stress of 35.5–36.4 MPa (Table 2), which were only slightly lower than that of parent SPEU film. The results indicated that the chemical treatments had minimum deterioration on the intrinsic properties of the substrate and the molecular weight of surface-grafted PEG had a marginal influence on the mechanical properties except for initial modulus. It seemed that the initial modulus decreased with the increment of molecular weight of PEG, which needs further studies. Obviously, the mechanical properties of surface-modified films could also meet the clinical requirements of long-term implant biomaterials.

### 3.4. Surface Hydrophilicity and Swellability

**Figure 6:**
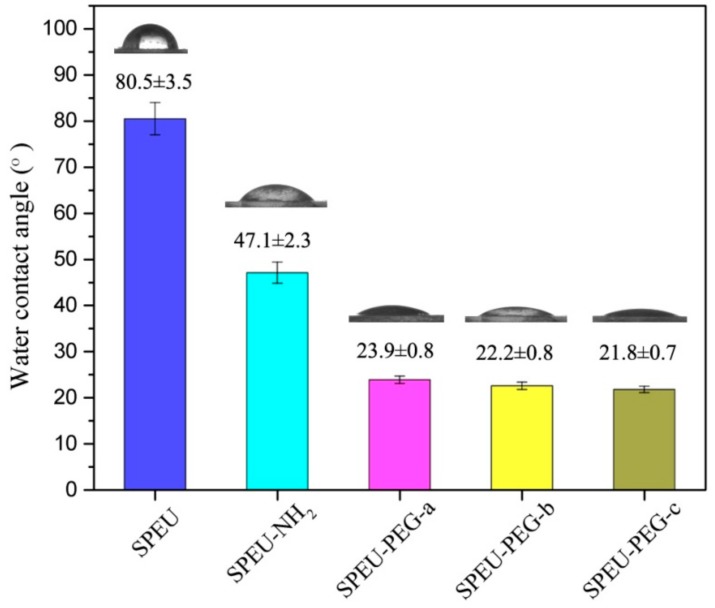
Water contact angle and images of the blank and modified SPEU films.

The surface hydrophilicity of the blank and modified SPEU films were characterized by sessile contact angle measurement, and the results are presented in Figure 6. The blank SPEU film exhibited a characteristic hydrophobic surface with a high-water contact angle of 80.5°, whereas the SPEU-NH_2_ film had low water contact angle of 47.1°, which indicated that the surface hydrophilicity had been improved after introducing -NH_2_ groups on surface. After the PEG was grafted onto the surface, the water contact angle decreased dramatically. The water contact angle of SPEU-PEG-a, SPEU-PEG-b, and SPEU-PEG-c was 23.9°, 22.2°, and 21.8°, respectively. It needs to be emphasized that the measure should be carried out immediately (within one s) after the ultrapure water is dripped onto the film surface, otherwise the water drop will spread out on the surface. The PEG grafted on the surface can interact with water molecules by the role of hydrogen bonding to form hydration layer on the film surface, resulting in low water contact angle. In addition, SPEU-PEGs exhibiting similar water contact angle indicated that the molecular weight of grafted PEG slightly affected the surface hydrophilicity, which should be due to the high grafting density of PEG on surface. These results supported that PEG had been grafted onto the SPEU surface and the hydrophilicity of PEG-grafted SPEU was improved significantly.

### 3.5. Protein Adsorption

**Figure 7:**
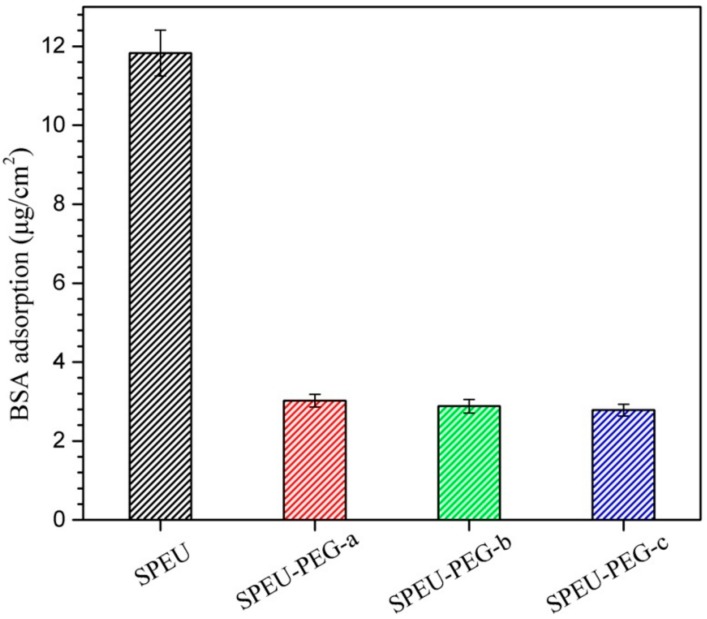
Adsorption behaviors of BSA on the surface of blank and PEG-grafted SPEU films at 37 ± 0.5 °C.

Protein adsorption on the surface of a material is always considered as the first step to evaluate the blood compatibility of the implanted or blood-contact biomaterials [37]. Figure 7 shows the adsorption behaviors of BSA on the surface of blank and SPEU-PEG films. The amount of adsorbed protein on the PEG-grafted surface (SPEU-PEG-a: 3.02 μg/cm^2^; SPEU-PEG-b: 2.88 μg/cm^2^; SPEU-PEG-c: 2.78 μg/cm^2^) was significantly lower than that of blank surface (SPEU: 11.87 μg/cm^2^), which was ascribed to the surface-grafted PEG. PEG chain not only has excellent hydrophilicity, but also possesses high flexibility. The highly flexible and well-hydrated chains can affect the fluidity of blood and hinder the protein adsorption on the surface [14]. The SPEU-PEGs exhibited similar protein adsorption quantity, indicating that the molecular weight of PEG grafted on the surface had marginal effect on the protein adsorption capacity. The result is inconsistent with that of PEG-grafted surface of commercial polyurethane (TT-1095A, Thermedics, Wilmington, MA, USA) in previous report [13], which should be due to the high grafting density of PEG on the surface. The lower protein adsorption capacity of SPEU-PEG surface means better surface hemocompatibility.

### 3.6. Platelet Adhesion

**Figure 8:**
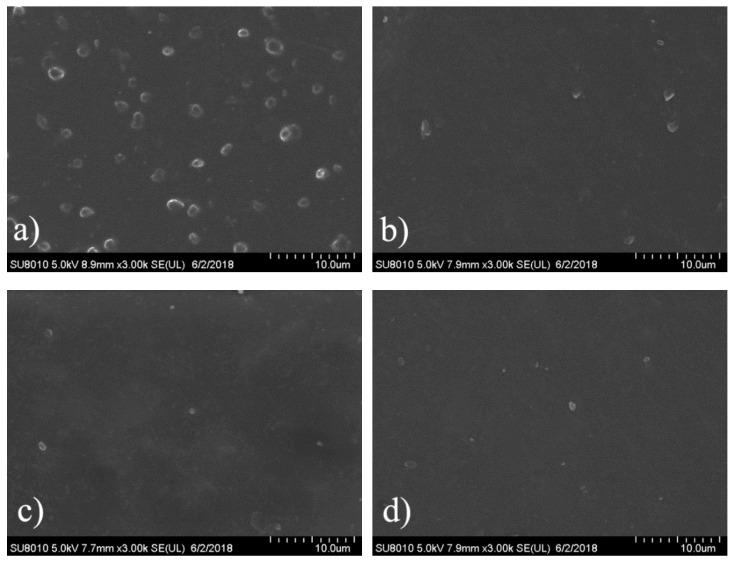
Representative SEM micrographs of platelet adhesion on the film surface of (**a**) SPEU; (**b**) SPEU-PEG-a; (**c**) SPEU-PEG-b; and (**d**) SPEU-PEG-c.

When blood is exposed to a foreign surface, initial protein adsorption on the surface is followed by platelet adhesion and release, which brings about cellular thrombogenesis [38]. Thus, platelet adhesion is rather important for the hemocompatibility of blood-contacting implantable materials. The morphologies of the platelets adherent on the surfaces of the blank and PEG-grafted SPEU films were assessed by FE-SEM observation, and the typical micrographs and quantification of adhered platelets are given in Figure 8 and Table 3, respectively. Massive platelets adhering on the surface of blank SPEU film (Figure 8a). Some of the platelets aggregated to some extent, some presented shape variation and some were spread on the surface, which indicated a highly activated state. After PEG was grafted on the surface, as shown in the Figure 8b–d, the amount of adhered platelets was reduced dramatically, and no obvious aggregation and deformation of platelets appeared, which proved a better anti-platelet adhesion surface. The possible explanation for this excellent anti-platelet adhesion is that the hydrophilic surface decreases the blood and plasma proteins interfacial energy on the PEG-grafted surface, which suppresses platelet adhesion [39]. The SPEU-PEG films having similar quantity of adhered platelets (Table 3) indicated that the molecular weight of PEG grafted on the surface marginally affected on the anti-platelet adhesion capacity. Due to the grafting density of PEG on the surface being relatively high, the denser short chain of PEG can form a layer coating the entire surface. Analogous effects have been reported for PEG-grafted silica surfaces by Alstine [40].

## 5. Conclusions

In the paper, PEG was grafted on the surface of SPEU film with high grafting density to improve surface hemocompatibility. The PEG-grafted SPEU (SPEU-PEG) was prepared by three-step chemical treatments (allophanate reaction, condensation reaction, and Michael addition reaction) under mild reaction conditions. The surfaces were characterized by FT-IR, ^1^H NMR, XPS, and water contact angle. The SPEU-PEGs displaying a much lower water contact angle than SPEU indicated that the hydrophilic PEG chains improved the surface hydrophilicity significantly. The mechanical properties of the SPEU-PEG films were slightly lower than that of parent film, verifying that the chemical treatments had minimum deterioration on the mechanical properties of the substrate. The low BSA adsorption quantity (2.78–3.02 μg/cm^2^) and good anti-platelet adhesion capacity (781–697 per mm^2^) revealed that SPEU-PEGs had improved surface hemocompatibility. Moreover, due to the high grafting density, the molecular weight of grafted PEG had marginal effect on the surface hydrophilicity and hemocompatibility. The PEG-grafted SPEU films possessed outstanding mechanical properties (strain at break: 866–884%; ultimate stress: 35.5–36.4 MPa) and good surface hemocompatibility, implying its high potential to be applied as long-term implants and blood-contacting biomaterials.

## Figures and Tables

**Table 1:** Surface elemental composition of the SPEU, SPEU-NH_2_, and SPEU-PEG films by XPS.

Films	Atomic Content/%		
	C1s	O1s	N1s
SPEU	78.2	18.7	3.1
SPEU-NH_2_	74.8	14.9	10.3
SPEU-PEG-a	72.1	25.7	2.2
SPEU-PEG-b	71.3	27.1	1.6
SPEU-PEG-c	70.2	28.4	1.4

**Table 2:** Mechanical properties of CPU films.

Films	Strain at Break (%)	Ultimate Stress (MPa)	Yield Strain (%)	Yield Stress (MPa)	Initial Modulus (MPa)
SPEU	896 ± 25	38.1 ± 2.1	46.7 ± 2.6	20.2 ± 1.2	43.3
SPEU-NH_2_	866 ± 16	35.7 ± 1.8	45.8 ± 2.1	19.3 ± 1.1	42.1
SPEU-PEG-a	873 ± 18	36.1 ± 1.4	52.7 ± 2.2	19.8 ± 0.9	37.6
SPEU-PEG-b	879 ± 21	36.4 ± 1.9	56.8 ± 2.0	20.1 ± 1.1	35.4
SPEU-PEG-c	884 ± 20	35.5 ± 1.7	58.7 ± 1.9	19.9 ± 1.3	34.0

**Table 3:** Quantification of platelets adhering to the blank and MPC-grafted PEU surface.

Films	SPEU	SPEU-PEG-a	SPEU-PEG-b	SPEU-PEG-c
Quantity of adhered platelets (per mm^2^)	20,702 ± 880	781 ± 56	731 ± 57	697 ± 52
